# Supplementary material for: Effectiveness of Botulinum Toxin on Pain in Stroke Patients Suffering from Upper Limb Spastic Dystonia
Source: Toxins (Basel). 2022 Jan 5;14(1):39. doi: 10.3390/toxins14010039 (PMC8780435; doi:10.3390/toxins14010039)
Supplement: Supplementary file 1 [file toxins-14-00039-s001.zip › toxins-1449960-supplementary.pdf]

Supplementary Materials: Effectiveness of Botulinum Toxin on Pain in Stroke Patients Suffering from Upper Limb Spastic Dystonia

Carlo Trompetto, Lucio Marinelli, Laura Mori, Luca Puce, Chiara Avanti, Elena Saretti, Giulia Biasotti, Roberta Amella, Filippo Cotellessa, Domenico Restivo and Antonio Currà

Table S1. Detail of patients' scores at T0

Patient	MAS					Posture			DAS				PNRS			
	ShA	FoF	FoP	WrF	FiF	Hefter's pattern	fingers	thumb	H	D	P	PT	Rest	ROMp	DN4	Pain site
**1**	0	3	2	3	3	3	1	3	3	3	3	2	6	8	1	shoulder
**2**	0	2	2	2	3	3	3	1	2	2	2	2	2	7	2	shoulder
**3**	0	1	1	0	2	3	3	1	1	2	1	1	2	3	0	wrist-hand
**4**	1	2	1	3	3	4	2	2	1	3	0	2	4	8	2	elbow
**5**	0	1	3	2	3	0	1	3	2	2	1	2	3	7	1	shoulder
**6**	3	3	2	2	3	5	2	2	3	3	2	1	6	8	4	elbow
**7**	0	1	3	4	1	4	0	0	2	2	2	1	8	10	4	wrist-hand
**8**	1	3	3	3	4	3	1	0	2	1	3	2	9	10	1	wrist-hand
**9**	3	4	4	4	3	4	3	0	2	2	2	2	2	9	2	elbow
**10**	0	2	0	0	4	3	2	2	2	2	0	1	0	3	0	wrist-hand
**11**	0	4	3	4	3	4	3	2	2	2	2	0	0	9	0	elbow
**12**	0	3	3	3	4	3	1	1	3	2	3	2	0	10	3	wrist-hand
**13**	0	4	3	4	0	4	0	0	1	2	0	1	0	3	0	wrist-hand
**14**	3	4	0	3	4	3	3	0	2	2	1	2	0	8	2	wrist-hand
**15**	0	1	4	4	4	4	1	1	2	2	1	2	0	4	0	elbow
**16**	0	2	0	3	4	3	1	1	2	2	0	1	0	8	0	wrist-hand
**17**	0	3	0	4	0	4	0	0	2	2	0	1	0	4	0	wrist-hand
**18**	2	2	3	4	0	4	0	2	0	3	0	0	0	7	0	shoulder
**19**	0	2	2	3	3	3	3	1	1	1	2	2	0	5	3	shoulder
**20**	0	1	2	3	2	3	2	1	2	3	0	1	0	2	2	wrist-hand
**21**	0	2	0	2	4	1	1	0	2	2	0	2	0	2	0	wrist-hand
**22**	1	1	0	0	4	0	1	0	3	1	2	1	0	10	0	wrist-hand
**23**	1	2	3	2	3	3	1	2	1	1	0	2	0	0	0	
**24**	0	1	1	0	2	0	1	3	1	1	0	0	0	0	0	
**25**	0	0	1	1	2	0	3	0	0	0	0	0	0	0	0	
**26**	0	0	0	2	4	1	1	3	2	1	0	1	0	0	0	
**27**	0	2	2	1	2	3	2	1	2	1	0	0	0	0	0	
**28**	0	3	2	2	3	3	1	1	2	1	0	1	0	0	0	
**29**	2	4	0	4	4	1	1	3	2	2	0	2	0	0	0	
**30**	0	1	1	2	3	3	2	2	3	2	0	1	0	0	0	
**31**	2	1	3	0	2	3	2	2	1	2	0	1	0	0	0	
**32**	0	1	3	2	3	3	1	3	1	2	0	2	0	0	0	
**33**	0	3	1	3	3	3	1	1	2	2	0	2	0	0	0	
**34**	0	1	2	3	4	4	1	1	2	2	0	1	0	0	0	
**35**	0	1	2	2	2	3	2	3	2	2	0	1	0	0	0	
**36**	0	2	3	4	4	4	1	1	2	2	0	1	0	0	0	
**37**	0	1	3	1	3	3	3	0	1	3	0	3	0	0	0	
**38**	2	1	1	2	3	3	3	1	1	2	0	2	0	0	2	

**39**	0	2	0	0	3	3	2	0	2	2	0	2	0	0	0
**40**	0	1	1	0	3	3	2	0	2	2	0	1	0	0	0
**41**	2	3	4	3	3	3	1	1	2	2	0	1	0	0	1

MAS Modified Ashworth Scale; DAS Disability Assessment Scale; PNRS Pain Numeric Rating Scale; ShA: shoulder adductors; FoF: forearm flexors; FoP: forearm pronators; WrF: wrist flexors; FiF: finger flexors. H: hygiene; D: dressing; P: pain; PT: posture. ROMp: Range of Motion passive. DN4: questionnaire of neuropathic pain. In red fonts scores in not-injected muscles.

Table S2. Detail of patients' scores at T1.

Patient	MAS					Posture			DAS				PNRS	
	ShA	FoF	FoP	WrF	FiF	Hefter's pattern	fingers	thumb	H	D	P	PT	Rest	ROMp
**1**	0	1	2	3	3	0	2	3	2	2	1	2	0	3
**2**	0	1	0	0	1	2	0	0	2	2	2	2	1	5
**3**	0	1	1	0	2	3	0	0	1	2	0	1	0	0
**4**	1	0	0	0	1	3	0	2	1	1	0	1	0	0
**5**	0	1	2	0	0	0	0	2	1	1	0	1	0	3
**6**	2	3	2	1	3	5	2	2	3	3	2	1	1	9
**7**	0	0	0	2	2	4	0	0	0	0	0	0	0	6
**8**	1	2	1	2	4	3	2	0	0	1	0	0	0	4
**9**	3	4	4	3	2	4	3	0	1	2	2	2	0	9
**10**	0	0	0	0	3	0	2	0	1	2	0	0	0	0
**11**	0	3	3	3	3	4	3	2	1	1	0	0	0	8
**12**	0	0	2	0	3	0	2	0	2	1	3	2	0	7
**13**	0	3	2	4	0	4	0	0	1	2	0	1	0	0
**14**	2	2	0	3	3	3	3	0	1	2	0	0	0	4
**15**	1	1	2	2	3	4	2	1	1	2	1	2	0	7
**16**	0	0	0	2	3	0	2	0	1	1	0	0	0	0
**17**	0	1	0	3	0	4	0	0	1	1	0	0	0	4
**18**	1	1	3	3	0	4	0	0	0	3	0	0	0	5
**19**	0	1	0	0	0	0	0	0	0	0	1	1	0	5
**20**	0	1	0	0	3	0	2	0	1	2	0	1	0	0
**21**	0	2	0	2	4	1	1	0	2	2	0	2	0	2
**22**	1	1	0	0	3	0	1	0	2	1	1	1	0	6
**23**	1	1	3	1	2	0	0	0	1	0	0	1	0	0
**24**	0	1	0	0	0	0	0	2	0	1	0	0	0	0
**25**	0	1	0	0	0	0	0	2	0	0	0	0	0	0
**26**	0	0	0	0	2	1	0	2	2	1	0	1	0	0
**27**	0	2	0	0	0	3	3	0	1	1	0	0	0	0
**28**	0	1	1	0	0	3	0	0	1	1	0	0	0	0
**29**	2	3	0	1	3	1	2	0	1	1	0	2	0	0
**30**	0	0	0	0	0	3	0	0	2	1	0	1	0	0
**31**	1	0	1	0	2	3	2	0	1	2	0	0	0	0
**32**	0	1	2	1	3	3	2	2	1	2	0	1	0	0
**33**	0	0	0	0	0	0	0	0	1	1	0	1	0	0
**34**	0	1	2	2	3	4	2	0	2	2	0	1	0	0
**35**	0	0	0	0	2	3	2	2	2	2	0	0	0	0
**36**	0	2	0	2	3	4	2	0	2	2	0	1	0	0
**37**	0	0	0	0	2	0	3	0	0	3	0	2	0	0
**38**	1	0	0	0	1	3	0	0	1	2	0	1	0	0
**39**	0	0	0	0	1	0	0	0	1	2	0	2	0	0
**40**	0	0	0	0	0	3	0	0	2	2	0	1	0	0
**41**	2	1	3	3	2	3	2	0	2	2	0	1	0	0

MAS Modified Ashworth Scale; DAS Disability Assessment Scale; PNRS Pain Numeric Rating Scale; ShA: shoulder adductors; FoF: forearm flexors; FoP: forearm pronators; WrF: wrist flexors; FiF: finger flexors. H: hygiene; D: dressing; P: pain; PT: posture. ROMp: Range of Motion passive. In red fonts scores in not-injected muscles.
